# Investigating the Role of Stress-Preventive Leadership in the Workplace Hospital: The Cross-Sectional Determination of Relational Quality by Transformational Leadership

**DOI:** 10.3389/fpsyt.2019.00622

**Published:** 2019-09-03

**Authors:** Felicitas Stuber, Tanja Seifried-Dübon, Monika A. Rieger, Contributors of the SEEGEN Consortium, Eva Rothermund, Stephan Zipfel, Harald Gündel, Florian Junne

**Affiliations:** ^1^Department of Psychosomatic Medicine and Psychotherapy, Medical University Hospital Tübingen, Tübingen, Germany; ^2^Institute of Occupational and Social Medicine and Health Services Research, University Hospital Tübingen, Tübingen, Germany; ^3^Department of Psychosomatic Medicine and Psychotherapy, Ulm University Medical Center, Ulm, Germany

**Keywords:** transformational leadership, relationship quality, health care sector, staff members, leaders

## Abstract

**Introduction:** A good relationship quality between leaders and staff members promotes mental health and prevents stress. To improve the relationship quality, it is important to identify variables which determine relationship quality at the workplace. Therefore, this study aims to identify specific leadership characteristics which support the development of a positive relationship between hospital leaders and staff members.

**Methods:** A cross-sectional study design was applied. A total number of 1,137 leaders (*n* = 315) and staff members (*n* = 822) of different professions (physicians, nursing staff, therapeutic professionals, administration staff, IT staff, clinical services, office assistants, scientists, others) working at a tertiary hospital in Germany assessed transformational leadership style as a staff-oriented leadership style and leader–member relationship quality by self-report questionnaires [integrative leadership questionnaire (FIF), leader–member exchange (LMX-7) questionnaire]. The data were statistically analyzed by mean comparisons and a multiple linear regression analysis.

**Results:** Leaders rated their own transformational leadership style (M = 3.98, SD = 0.43) systematically higher than staff members assessed their leader (M = 2.86, SD = 1.04). Evaluation of relationship quality showed similar results: leaders evaluated their relationship quality to one exemplary staff member higher (M = 4.06, SD = 0.41) than staff members rated their relationship quality to their direct leader (M = 3.15, SD = 0.97). From the staff members’ perspective, four sub-dimensions of transformational leadership, that is, “individuality focus,” “being a role model,” “fostering innovations,” and “providing a vision” showed large effect sizes in the regression analysis of relationship quality (*R* *^2^* = 0.79, *F* (14,690) = 189.26, *p* < 0.001, *f* = 1.94).

**Discussion:** The results of our study are in line with previous investigations in other working contexts and point to a profession-independent association as the professional group of participants did not contribute to the variance explanation of the regression analysis. The exploration of potential determinants of relationship quality at work can, for example, support the development of leadership training programs with a focus on transformational leadership style. This might be an opportunity to foster high relationship quality between leaders and staff members and consequently might represent one strategy to prevent stress in the health care sector.

## Introduction

Considering the maintenance of employees’ mental health as an operational task, and thus as a leader’s task, has indeed an ethical aspect and is also a legal imperative in Germany. In 2012, the legal obligation of German employers to assess and reduce psychological health risks at the workplace was substantiated by an amendment of the respective German occupational health and safety act (1). Accordingly, the employer has to judge the risk to which employees are exposed to at their workplace including psychological stress at work and to determine which measures of occupational safety and health are necessary to reduce this risk. With regard to psychological stress at work, working conditions as well as social relationships (e.g., workplace bulling and harassment) and the working culture have to be addressed (2), with leadership being one important aspect.

Empirically, leadership has been found to be an important variable in relation to job performance (3, 4) as well as employees’ health (5, 6). That is, different leadership styles are differentially associated with employees’ job performance and mental health. Destructive leadership is defined as a deleterious behavior against a person and/or an organization in an active or passive way (7). It reduces productivity and has detrimental effects on the health of staff members (8), whereas appreciative leadership behavior leads to a higher work satisfaction (9), higher intention to stay at the present workplace (10), and higher well-being of staff members (11–13) as well as to improvements in leaders’ own well-being (14).

A unifying characteristic of all these staff-oriented leadership behaviors is the importance of the relationship between leaders and staff members. A leadership approach that elaborates on this dyadic relation between direct leaders and their staff members is the leader–member exchange (LMX) approach [for an overview, see (15)]. The LMX approach targets the specific and individual dyad between one leader and one staff member. Thus, relationship quality between a leader and his/her various staff members can differ (16) and the development of the dyadic relationship can be described as a continuous process [e.g., Refs. (15, 17)].

A mature relationship has been positively related to several positive health and performance-oriented outcomes for staff members: for example, job performance (18), procedural distributive justice (19), and general job satisfaction (20). On the other hand, mature relationships were negatively related to turnover intention and role conflicts at work (3). Consequently, a mature relationship between leader and staff member is preferable at the workplace, although high relationship quality can be perceived as a rather abstract construct without clear recommendations on how to establish such relationships on a behavioral basis (21). Thus, research has tried to reveal factors that contribute to a mature leader–staff member relationship at the workplace on the part of staff members and leaders (3, 21). Although good relationships at the workplace are not only stress preventive for staff members but also for leaders, we decided to concentrate in this study on stress preventive implications for staff members (22).

In this study, we focused on behavioral leadership characteristics which have been found to be subject to change (23) and could explain a substantial variance of the quality of the leader–member relationship (3). The leadership style that has been found to be associated with mature leader–member relationships (3) is known as transformational leadership (24). Transformational leadership behavior is an appreciative and toward personal growth-oriented leadership style aiming to motivate staff members through, for example, long-term aims and adjustment of values. It supports staff members to focus not only on individual goals but also on group and organizational goals (25). Transformational leadership comprises six different core behaviors (25–27), which have been labelled as “fostering innovation,” “team spirit development,” “performance development,” “individuality focus,” “providing a vision,” and “being a role model” (28).

Empirically, transformational leadership behavior shows robust relations to performance-oriented and health-oriented outcomes. Specifically, transformational leadership is associated with increased job performance (27, 29), work-related satisfaction, and motivation [e.g., Refs. (29, 30)], attachment to the leader (31), fewer days of absence due to sickness, and fewer critical incidents at the hospital [e.g., Ref. (32)], as well as less perceived stress and higher well-being [e.g., Refs. (33–36)].

Although the association of transformational leadership in general with improved quality of the leader–member relationship (LMX) seems well supported by the current literature (37–39), the specific sub-dimensions of the transformational leadership approach that foster the quality of the leader–member relationship have not been well researched to date. Furthermore, evidence is lacking especially with regard to specific working contexts and professional groups, such as at the workplace hospital.

To explore determinants that could be associated with higher relationship quality between leaders and staff members at the workplace hospital seems to be an important point as relationship quality between the direct leader and her staff members is one of the few working conditions which can be influenced by leaders and staff members themselves and therefore constitutes an opportunity for stress prevention (33). As the workplace hospital is a psychologically demanding workplace where studies showed an increasing burnout and depression level in physicians (40) and where chronical work overload was also associated with poorer patient care (41, 42), maintaining psychological health, e.g., by strengthening relationship quality is of particular importance. Although professional groups within the workplace hospital differ in their every day work, they are unified by the fact of social interaction and relationships between leaders and staff members. Thus, further research is needed to clarify the specific determinants, as part of the transformational leadership behavior, that lead to improved leader–member relationships at this specific organization [for an overview on the relevance of context see Ref. (43)].

Therefore, this study was conducted in the context of a tertiary hospital in Germany to examine the association between transformational leadership sub-dimensions with the quality of the perceived leader–member relationship.

The study aims to answers the following research questions:

1. How do leaders perceive the quality of their relationship with staff members and vice versa?
2. Does the perception of transformational leadership differ between leaders and staff members?
3. In which way are the sub-dimensions of transformational leadership behavior associated with the quality of leader–member relationships from the view of staff members in the workplace hospital?

## Materials and Methods

### Implementation

A cross-sectional online survey was conducted from May 23, 2018, to July 18, 2018, and was approved by the ethics committee of the University Hospital and Medical Faculty of Tübingen (622/2017BO2) as well as by the chief executive board and the employees’ council of the tertiary hospital. Completion time for the online survey was about 10 min. Overall, *N* = 10,101 employees received the survey invitation and the response rate was 11.26%.

### Materials

We created an online survey with questions on transformational leadership behavior and relationship quality using validated standardized instruments delivered *via* the Unipark survey software (QuestBack GmbH). Questions on both aspects were asked either from the leaders’ or the staff members’ perspective. That is, leaders evaluated their own leadership behavior, whereas staff members assessed their direct supervisor. To discriminate participating employees according to their hierarchy level, employees had to define themselves either as leaders or as staff members. Yet, there was no possibility to assess leaders and their directly associated team due to data protection requirements.

#### Questionnaire Assessing Transformational Leadership

**Figure 1:**
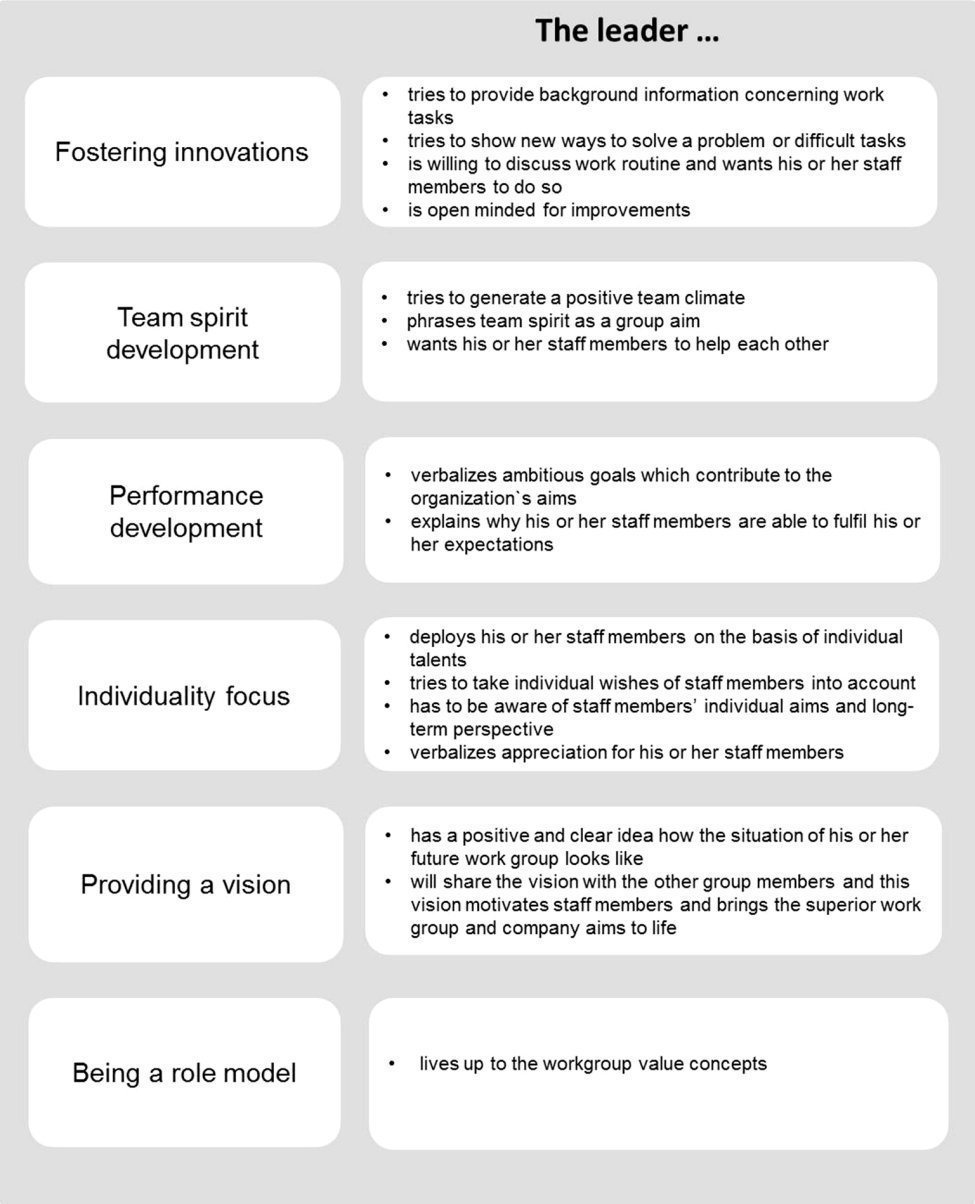
Description of transformational leaderships sub-dimensions (translated by the authors from the description by 28, pp. 8–9).

The questionnaire used to assess the sub-dimensions of transformational leadership was the “integrative leadership” questionnaire (Fragebogen zur Integrativen Führung, FIF) (28), a standardized instrument which measures leadership and communication style in four modules. In our survey, we applied transformational leadership as one part of the “integrative leadership” questionnaire. The construct of transformational leadership in the questionnaire draws on the concept of Heinitz and Rowold (26) and Ref. (25), see **Figure 1** for more details). Participants were asked to rate 32 statements using a five-point Likert scale from 1 (agree not at all) to 5 (totally agree). The item ratings can be summarized in six different scale scores or in one overall transformational leadership score. The scales of transformational leadership show a sufficient internal consistency with Cronbach’s α = 0.83–0.92 for the staff members’ assessment provided by the manual (28) and Cronbach’s α = 0.86–0.94 for the staff members’ assessment by our study. In addition, Cronbach’s α = 0.75–0.83 for the leaders’ assessment provided by the manual (28) and Cronbach’s α = 0.67–0.81 for the leaders’ assessment by our study. The convergent validity of the transformational leadership scale of the FIF was confirmed by high correlations with the frequently used questionnaire Transformational Leadership Inventory (TLI) (25, 26).

#### Questionnaire Assessing the Quality of the Leader–Member Relationship

The LMX-7 questionnaire (15, 44) in its German version is based on the LMX model (15) which represents the relationship quality between leaders and staff members. It is a standardized unidimensional scale with seven items. Participants are asked to rate seven questions and statements on a five-point Likert scale from 1 (low relationship quality) to 5 (high relationship quality) either in a version for leaders to assess the relationship quality to one exemplary staff member or in a version for staff members to assess the relationship quality to their direct leader. Graen and Uhl-Bien (15) postulated that the LMX-7 measures the three highly correlated relationship aspects respect, trust, and obligation as one LMX dimension. The ratings of the participants can be summarized and presented through one overall LMX score. The LMX-7 has shown high internal consistency for staff members’ ratings (Cronbach’s α = 0.89 and α = 0.92), whereas internal consistence was not reported for leaders’ rating (44). In our study, LMX-7 showed an internal consistency of α = 0.74 for leaders and α = 0.93 for staff members.

### Statistical Analyses

For the description of the participants as well as for descriptive specifications of leadership behavior and relationship quality, mean (M), percentage (%) and distribution in the form of standard deviation (SD) were applied. To compare leaders’ and staff members’ ratings, we used *t*-tests as the data satisfied the condition of normal distribution. To determine the effect size of mean comparisons, Cohen’s d was applied. A result of *d* ≤ 0.2 can be interpreted as a small, *d* ≤ 0.5 as a medium, and *d* ≤ 0.8 as a large effect size (45). Moreover, a multiple linear regression was conducted to explore the association between transformational leadership subdimensions and LMX overall score. Assumptions of multiple regressions (linearity, normality, homoscedasticity, and independence of residuals) were checked, and *f* was reported for the effect size. A result of *f* ≤ 0.10 can be interpreted as a small, *f* ≤ 0.25 as a medium and *f* ≤ 0.40 as a large effect (46). The level of significance was set for all analyses to α = 0.05, and all analyses were conducted by using IBM SPSS version 25. For multiple comparisons, we adjusted alpha levels by Bonferroni correction. Total scores of transformational leadership behavior and relationship quality were only calculated when no missing values occurred in sub-dimensions. Concerning the linear multiple regression, cases were only included when no values of subdimensions and total scores were missing. As the variable *Professional Group* was categorial with the categories: physicians, nursing staff, therapeutic professionals, administration staff, IT staff, clinical services, office assistants, scientists, and other professions, dummy coding was used for the linear multiple regression. For the baseline group, the category Administration staff was chosen as this professional group was the largest. A dummy variable is defined in our linear multiple regression as the difference in relationship quality perception for the administration staff and one other professional group [either physicians or nursing staff or therapeutic professionals or IT staff or clinical services or office assistants or scientists or other professions; for a detailed description of dummy coding, see Ref. (47), p. 208–215].

## Results

### Population

A total of 1,137 employees of a tertiary hospital in Germany participated in the study, with 315 (27.7%) identifying themselves as leaders and 822 (72.3%) as staff members without leadership responsibilities. Of the staff members, 554 (74.8%) were female and 187 (25.2%) were male, whereas in the leader group 174 (59.6%) were female and 118 (40.4%) were male. One hundred four participants provided no information on their gender. For detailed information on the characteristics of the participants, see **Tables 1**–**2**.

**Table 1:** Age group frequencies depending on hierarchy level.

Age groups in years	Hierarchical group			
	Staff members		Leaders	
	*%*	*n*	*%*	*n*
<20–24	3.7	30	0.3	1
25–30	15.1	123	3.5	11
31–35	11.2	91	9.3	29
36–40	12.6	103	12.2	38
41–45	8.6	70	15.1	47
46–50	13.7	112	13.5	42
51–54	15.1	123	17.6	55
>55	20.1	164	28.5	89

%, percent; n, number of participants; n = 6 staff members and n = 3 leaders didn’t provide information on their age, N = 1128.

**Table 2:** Proportion of professional groups depending on hierarchy level and depending on professional groups overall.

Professional groups	Hierarchical level				Overall	
	Staff members		Leaders			
	*%*	*n*	*%*	*n*	*%*	*n*
Physicians	53.8	84	46.2	72	13.7	156
Nursing staff	67.6	142	32.4	68	18.5	210
Therapeutic professionals^a^	80.8	59	19.2	14	6.4	73
Administration	70.4	157	29.6	66	19.6	223
IT	78.9	56	21.1	15	6.2	71
Clinical services^b^	72.7	8	27.3	3	1.0	11
Office assistants	89.3	100	10.7	12	9.9	112
Scientists	77.0	87	23.0	26	9.9	113
Others	76.8	129	23.2	39	14.8	168

%, percent; n, number of participants, N = 1137.

a e.g. physiotherapist, psychotherapist.

b e.g. caretaker service, catering.

### Transformational Leadership Behavior at the Hospital

Leaders (M = 3.98, SD = 0.43, n = 275) and staff members (M = 2.86, SD = 1.04, n = 737) differed significantly in their perception of the total transformational leadership score at their workplace [*t* (1,000.31) = −24.21, *p* < .001, *d* = 1.23]. Leaders assessed themselves as leading more transformational than the staff members evaluated their direct leaders. This result was seen for all sub-dimensions as well: leaders rated themselves in all dimensions higher than staff members evaluated their leaders (see **Table 3**). Expect one sub-dimension (performance development) which revealed a medium size effect, all other sub-dimensions showed a high effect size.

**Table 3:** Leaders’ and subordinates’ ratings of transformational leadership sub-dimensions.

Sub-dimensions of TFL	Staff members			Leaders					
	*M*	*SD*	*n*	*M*	*SD*	*n*	*t(df)*	*p*	*d*
Fostering innovations	3.15	1.10	811	4.27	0.50	307	(1,082.34) = −23.38	<.001	1.15
Team spirit development	2.84	1.18	805	4.07	0.60	304	(1,017.23) = −22.69	<.001	1.17
Performance development	2.89	1.09	794	3.66	0.73	300	(799.81) = −13.30	<.001	0.77
Individuality focus	2.78	1.22	805	4.02	0.61	307	(1,038.91) = −22.41	<.001	1.14
Providing a vision	2.55	1.17	798	3.57	0.74	306	(872.58) = −17.26	<.001	0.96
Being a role model	3.00	1.30	803	4.35	0.55	299	(1,085.18) = −24.26	<.001	1.18

TFL, transformational leadership; M, mean; SD, standard deviation; n, number of included participants; t, t-test statistic; df, degrees of freedom; p, p-value; d, Cohen’s d.

### LMX at the Hospital

Leaders and staff members perceived the relationship quality between leaders and staff members at the hospital in significantly different ways [*t* (1054.83) = −21.68, *p* < .001]. Leaders (M = 4.06, SD = 0.41, n = 293) rated the relationship quality they offered to one exemplary staff members higher than the subordinates rated their relationship quality with their direct leaders (M = 3.15, SD = 0.97, n = 777).

### Sub-Dimensions of Transformational Leadership as Potential Determinants of Relationship Quality from a Staff Members’ Perspective

vLinear multiple regression analysis was applied to assess the extent to which the sub-dimensions of transformational leadership behavior determine the variance of the perceived relationship quality at the hospital from a staff members’ perspective. Professional groups of the staff members (see **Table 2**) were also entered as dummy variables into the linear multiple regression to control potential professional related differences in the association of transformational leadership and relationship quality. All assumptions of multiple regression analysis were met, and predictors were all entered simultaneously into the model (see **Table 4** for correlations of continuous variables). The result of the linear multiple regression analysis is presented below in **Table 5**.

**Table 4:** Intercorrelations of transformational leadership sub-dimensions and relationship quality from a staff members’ perspective.

Variables	1	2	3	4	5	6	7
1. Staff members’ total LMX	—	0.78***	0.76***	0.66***	0.84***	0.77***	0.80***
2. Fostering innovations		—	0.78***	0.70***	0.75***	0.77***	0.77***
3. Team spirit development			—	0.71***	0.74***	0.74***	0.78***
4. Performance development				—	0.62***	0.75***	0.70***
5. Individuality focus					—	0.74***	0.73***
6. Providing a vision						—	0.78***
7. Being a role model							—

Pearson correlations for staff members (n = 705) are presented above the diagonal. *** p < .001.

**Table 5:** Linear multiple regression analysis for staff members’ perception of relationship quality.

Sub-dimensions	*B*	*SE(B)*	β	*t*	*p*	*CI(B)*
Constant	0.93	0.06	—	14.49	<0.001	0.80–1.05
Admin. vs Physicians	−0.11	0.07	−0.04	−1.70	0.09	−0.24 to 0.02
Admin. vs nursing staff	−0.12	0.06	−0.04	−1.92	0.06	−0.22 to 0.00
Admin. vs Therapeutic professionals	−0.02	0.07	−0.06	−0.32	0.75	−0.17 to 0.12
Admin. vs IT staff	−0.02	0.08	−0.01	−0.24	0.81	−0.18 to 0.13
Admin. vs Clinical services	0.11	0.23	0.01	0.50	0.62	−0.34 to 0.56
Admin. vs Office assistants	−0.08	0.06	−0.03	−1.24	0.22	−0.20 to 0.05
Admin. vs Scientists	−0.04	0.07	−0.01	−0.056	0.58	−0.17 to 0.09
Admin. vs Other professions	0.04	0.06	0.02	0.69	0.49	−0.07 to 0.15
Fostering innovation	0.11	0.03	0.13	3.66	<0.001	0.05–0.17
Team spirit development	0.05	0.03	0.07	1.96	0.05	0.00–0.11
Performance development	0.02	0.03	0.02	0.63	0.53	−0.03 to 0.07
Individuality focus	0.35	0.02	0.43	14.52	<0.001	0.30 to 0.39
Providing a vision	0.08	0.03	0.09	2.62	<0.01	0.02 to 0.13
Being a role model	0.19	0.03	0.25	7.55	<0.001	0.14 to 0.24

B, unstandardized coefficient; SE, standard error of B; β, standardized coefficient Beta; t, t-test; p = p-value; CI, confidence interval of B, n = 705 subordinates; Admin., Administration staff, R^2^ = 0.79, F (14,690) = 189.26, p < .001.

The total variance of relationship quality that could be explained by this model was 79% [*R* *^2^* = 0.79, *F* (14,690) = 189.26, *p* < .001] which corresponded to a large effect (*f* = 1.94). The sub-dimensions “fostering innovation,” “individuality focus,” “providing a vision,” and “being a role model” were included as significant determinants of the variance explanation. Standardized beta values (β) revealed that on a single factor level the sub-dimensions “individuality focus” and “being a role model” made the strongest contribution to explain the variance of relationship quality.

## Discussion

To our knowledge, this is the first study which investigates sub-dimensions of transformational leaderships and the quality of leader–member relationships across all professions in the workplace hospital from leaders’ and staff members’ point of view. Leaders and staff members’ perception of transformational leadership and relationship quality at the workplace hospital differed significantly on an overall basis and at a dimensional level. That is, leaders rated transformational leadership behavior and relationship quality higher than the staff members of the same hospital did. Furthermore, the results provide insight into the association between the sub-dimensions of transformational leadership and relationship quality from a staff members’ point of view: The sub-dimensions “individuality focus,” “being a role model,” “fostering innovations,” and “providing a vision” explained 79% of the variance of the perceived relationship quality, whereas the professional group of staff members could not contribute to the variance explanation.

When comparing our rating results of transformational leadership to the results of a representative sample of German leaders and subordinates provided by the manual of the questionnaire of integrative leadership (FIF) (28), the ratings of our sample can be located in the lower half of the average range. That is, transformational leadership was perceived as average in our sample with a tendency to lower staff members’ ratings.

Relationship quality has been examined with the here used questionnaire LMX-7 in the health sector before (48). Research showed scale values for staff members’ perception of LMX relating to their direct leader in the medium range between 3.34 and 3.36 (21) and 3.32 (49). Our results are comparable to these study results with the tendency to lower staff members’ ratings parallel to the ratings of transformational leadership. Although our rating results seem at least comparable to other study results, taking into account relationship qualities’ impact on staff members’ well-being (50) and the potential improvement through transformational leadership with regard to fewer undesirable patient outcomes (e.g., medication errors), more job satisfaction (30), and higher occupational and patient safety culture in hospitals (51) an increasing rate of transformational leadership behavior and relational quality might be seen as desirable for the workplace hospital.

According to the rating discrepancy between leaders and staff members previous studies discussed that employees tended to rate their job performance more positively and less variably in self-assessments compared with other sources (e.g., peers, supervisors, subordinates) because of more indulgence and less discriminant validity (52). This result seems in line with our findings where leaders rated their transformational leadership behavior more positively and had less variance in their assessments than staff members showed in their ratings of transformational leadership behavior of their direct leaders. The ratings of the participating leaders in our sample could be contaminated by social desirability, similar to the results of Sarros et al. (53) who found significant correlations between personality characteristics (e.g. courage, compassion) and social desirability in leaders’ self-assessments.

Aside from this potential bias, it is worthwhile to discuss the meaning of such different perceptions of leaders and staff members concerning transformational leadership on an organizational level. Aarons et al. (54) interpreted these different perceptions as clues to the organizational culture quality. The results of their study showed an association between transformational leadership rating and organizational culture: the higher the rating discrepancy between leaders and staff members, the worse the organizational culture was, especially when leaders rated themselves as better than their staff members did. This shows the need to shorten the rating distance between leaders and staff members, although leaders’ and staff members’ rating cannot be related to each other directly.

To get a better understanding of what leaders can contribute to relationship quality from a staff members’ perspective, we ran a regression analysis with the result that four sub-dimensions of transformational leadership behavior (“individuality focus,” “being a role model,” “fostering innovations,” and “providing a vision”) significantly determined the relationship quality between leaders and staff members, whereas the professional group of the staff members did not contribute to the variance explanation. These findings may support the theoretical assumptions and empirical approaches of previous research that transformational leadership is associated positively with the LMX model (15, 20, 24, 38).

To discuss and classify the impact of the four sub-dimensions of transformational leadership on relationship quality a comparison to other study results concerning the dimensions “individuality focus,” “being a role model” and “providing a vision” is possible whereas the dimension “forstering innovation” has not been found to determine relationship quality before. That is, the explanation for the impact of the dimension fostering innovation is rather speculative. The effect of the dimension “fostering innovation” could be explained by the health care sector as study context: Employees working there could show a higher affinity to innovations in general as improving patient care through innovative treatment methods can be seen as one important part of medical advance which is important for employees’ every day work in the health care sector. Although the association of “fostering innovations” and relationship quality has not been explained explicit yet, this dimension has been associated significantly positive to other staff-oriented variables like job satisfaction, affective commitment and organizational citizenship behavior (28).

The dimensions “individuality focus” and “being a role model” could explain a considerable higher part of variance than “fostering innovations” in the performed regression analysis. Both aspects could be seen as a part of high employee orientation and are in line with other empirical approaches. Deluga (55) examined the relationship of transformational leadership and relationship quality on a sub-dimensional level as well. He found on the basis of the four factorial transformational leadership model (56) the sub-dimension “charisma” [corresponding to parts of the sub-dimension “providing a vision” and “being a role model” in our study; see Ref. (28)] and “individual consideration” [corresponding to the sub-dimension “individuality focus” in our study; see Ref. (28)] as two predictors for relationship quality in the military context. Yukl et al. (57) showed in their study that the transformational leadership sub-dimensions “leading by example” [corresponding to the sub-dimension “being a role model” in our study; see Ref. (28)] could explain parts of the variance of relationship quality.

Our results revealed comparable sub-dimensions of transformational leadership related to relationship quality for the hospital context as Deluga (55) found for the context of the U.S. Navy. This concordance has been shown despite very different working contexts and thereby could lead to the assumption that the relation of transformational leadership subdimensions and relationship quality could be quite independent of the working context. The idea of generalization is also supported by the result of our regression analysis that the professional group of staff members did not contribute to the variance explanation of relationship quality. The association between transformational leadership and relationship quality is independent of the professional group in our study. Future investigations could examine this aspect further by including first and secondary care hospitals or focusing on other sectors. For example, the economic sector where leaders have more direct access to monetary resources, as studies have shown that transformational leadership style is especially relevant when leaders have no direct access to monetary reward systems (29) and when workplaces are more hierarchically structured (36), which are both applicable for our study as well as for Deluga’s (55) study context but won’t fit to the economic sector in the same way.

Further research is needed to investigate the effect level of sub-dimensions of transformational leadership behavior (e.g. individual level, dyadic level, group level or organizational level). Seltzer and Bass (58) assumed that the sub-dimension “charisma” and thus also the sub-dimension e.g., “providing a vision” mainly have an effect on a dyadic level as well as the outcome variable relationship quality. We assume that “individuality focus” and “being a role model” could also show an effect on a dyadic level as they can be perceived as the relationship-based sub-dimensions of transformational leadership.

### Limitations

First, ratings of leaders and staff members cannot be associated directly with each other (the leaders rated by staff members might not be the ones that have participated in the study). That is, it could be possible that the most transformational leaders and the most unsatisfied staff members participated and distorted the survey results in the respective directions. Future studies should aim to enable the connection between a leader’s self-ratings and the ratings of their actual respective staff members. Second, future investigations need to use more than just one measurement method (e.g., self report questionnaires and qualitative data from outside observers). As the exclusive use of self-report tools is an important limitation of our study. Third, we had a low response rate, and participation in our survey was voluntary, which may also have rendered the sample less representative with, for example, the more motivated employees participating. Fourth, the cross-sectional design hinders causal inference from the study results but gave the opportunity to consider the relation of transformational leadership sub-dimensions and relationship quality without adding any temporal variables in this early stage of study (59). Another point is the high proportion of variance explanation in the regression analysis which could be a hint for overestimation of the relation between the sub-dimensions of transformational leadership and relationship quality although the two constructs can be distinguished by their theoretical background: Whereas transformational leadership focuses on leadership behavior, the model of relationship quality refers to the relationship between leaders and staff members. Despite this potential overestimation, the investigated association can be seen as one important part of relationship quality research besides other examined determinants like subordinates’ characteristics, interactional characteristics, and context variables (60).

To sum up, the hypotheses that can be raised from our results may well justify future studies that employ interventional longitudinal designs to enlighten the effects of transformational leadership on relationship quality as well as the by now theoretical based assumption that there is an opportunity to prevent stress by fostering relationship quality.

### Practical Implication

This study explored specific determinants of relationship quality in the workplace hospital to explore opportunities to enhance relationship quality. Based on our results first, leaders should remember that their transformational leadership behavior could have an impact on the relationship quality with their staff members. And that by fostering the relationship quality, an opportunity to prevent stress in their staff members comes along. Second, leaders should get the opportunity to participate in leadership training programs to reflect, develop, and improve their transformational leadership skills. Studies have already shown that transformational leadership can be improved by leadership interventions (61, 62).

The next step should be to assess whether this can lead to a change in perceived relationship quality as an important working condition regarding staff members’ well-being in the health care sector. Besides other important measures (e.g. reduction of high quantitative demands, improving personnel shortage, addressing the hazardous of working with critical ill patients), this ultimately might represent one of the promising strategies to prevent stress-related disorders in the health workforce.

## Contributors of the SEEGEN Consortium

Eva Rothermund, Nadine Mulfinger, Mark Jarczok, Department of Psychosomatic Medicine and Psychotherapy Ulm University Medical Center, Ulm, Germany; Peter Angerer, Institute of Occupational and Social Medicine University Hospital Düsseldorf, Düsseldorf, Germany; Imad Maatouk, Clinic of General Internal Medicine and Psychosomatic, Heidelberg, Germany; Andreas Müller, Faculty of Educational Science Work and Organizational Psychology, Essen, Germany; Bernd Puschner, Department of Psychiatry and Psychotherapy II Ulm University and Bezirkskrankenhaus Günzburg, Ulm, Germany; Jochen Schweitzer-Rothers, Institute for Medical Psychology University Hospital Heidelberg, Heidelberg, Germany; Stefan Süß, Department of Business Administration, in particular Work, Human Resource Management and Organization Studies Heinrich-Heine-University Düsseldorf, Düsseldorf, Germany; Ute Ziegenhain, Clinic of Child- and Adolescents Psychiatry/Psychotherapy University Hospital Ulm, Ulm, Germany.

## Data Availability

The implementation of this study had to be approved by the chief executive board and the employees’ council of the tertiary hospital. This approval required that raw data were only made available to direct project associates.

## Ethics Statement

The study was approved by the ethics committee of the University Hospital and Medical Faculty of Tübingen (622/2017BO2) named Ethik-Kommission an der Medizinischen Fakultät der Eberhard-Karls-Universität und am Universitätsklinikum Tübingen and was carried out in accordance with the recommendations of the ICH-GCP-guidelines, Declaration of Helsinki. All subjects gave written informed consent in accordance with the Declaration of Helsinki.

## Author Contributions

FS, TS-D, and FJ planned and conducted the study. MR, SZ and HG as well as the contributors of the SEEGEN Consortium gave feedback and support during the writing process of the manuscript.

## Funding

The study was partly funded by the Federal Ministry of Education and Research within the “SEEGEN”-study (FKZ 01GL1752C) and by the institutions own financial resources.

## Conflict of Interest Statement

The authors declare that the research was conducted in the absence of any commercial or financial relationships that could be construed as a potential conflict of interest.
